# Yeast Prions: Proteins Templating Conformation and an Anti-prion System

**DOI:** 10.1371/journal.ppat.1004584

**Published:** 2015-02-05

**Authors:** Reed B. Wickner, Herman K. Edskes, David A. Bateman, Anton Gorkovskiy, Yaron Dayani, Evgeny E. Bezsonov, Maryam Mukhamedova

**Affiliations:** Laboratory of Biochemistry and Genetics, National Institute of Diabetes and Digestive and Kidney Disease, National Institutes of Health, Bethesda, Maryland, United States of America; Washington University School of Medicine, UNITED STATES

## Introduction

Most yeast prions (infectious proteins) are amyloids, linear β-sheet-rich polymers of a single protein with the β-strands perpendicular to the long axis of the filament. A single prion protein can form any of many different prion variants, differing in structure and biological properties, but with the same amino acid sequence. The folded in-register parallel β-sheet architecture we have shown for several yeast prions explains how a given prion variant can be propagated stably, how a protein can template its conformation, just as DNA can template its sequence. We have found an anti-prion system that sequesters prion seeds, preventing their even distribution to daughter cells. This system involves Btn2p, Cur1p, and Hsp42, and cures most of the [URE3] variants arising. Btn2p is weakly homologous to mammalian HOOK proteins, which include the aggresome-active HOOK2.

While mammalian prions (infectious proteins) are rare, many common human amyloid diseases, such as Alzheimer disease, Parkinson disease, and others, have prion aspects, including infectivity in some cases. Most yeast prions are infectious amyloids, filamentous polymers of a single protein. Work on yeast prions was the first to establish the protein-only (prion) hypothesis, and, now, work on the structure of yeast prion infectious amyloid explains how a prion protein can template its conformation, just as DNA can template its sequence. The recent discovery of a cellular anti-prion system that cures most arising prions of the yeast Ure2 protein offers a possible direction to look for treatments of amyloidoses.

The yeast non-chromosomal genetic elements [URE3] and [PSI+] were discovered to be prions of Ure2p and Sup35p, respectively, based on their unusual genetic properties [1], and were demonstrated to be based on amyloids of these proteins [2–4]. There are now at least eight amyloid-based prions of *Saccharomyces cerevisiae* (reviewed in [5,6]) and one of *Podospora anserina* [7]. In addition to the PrP-related mammalian prion diseases, recent work indicates that Alzheimer disease and other common human amyloidoses have prion-like aspects, including infectivity (reviewed by [8]). This broadens the importance of detailed studies of yeast prions.

## Prion Variants

[PSI+] and [URE3] show a remarkably wide array of variants: strong and several degrees of weak variants differ in the intensity of the prion phenotype; stable and unstable (each of which can be strong or weak); transmissible (or not) to a given sequence polymorph of the prion protein (each of which can be strong or weak); curable (or not) by overproduction or deficiency of various chaperones; and lethal, toxic, or mild in its detrimental effects on cells [9–11].

## Biology of Yeast Prions

Mammalian prions are uniformly fatal, and common variants of [PSI+] and [URE3] are lethal or nearly so [11], but other, quite mild variants of the same prions are most often studied. [PSI+] and [URE3] are rare in wild strains [12,13] showing that even the mildest variants are a net detriment to their hosts. Whether even the mildest [PSI+] or [URE3] confer any benefit in any strain is controversial, but the [HET-s] prion of *P. anserina* is beneficial to its host (reviewed by Saupe [7]). The ability of Ure2p and Sup35p to form the prions [URE3] and [PSI+] is not conserved but is found sporadically distributed among the species examined so far (e.g., [14]).

## Structure Explains Inheritance

**Fig 1:**
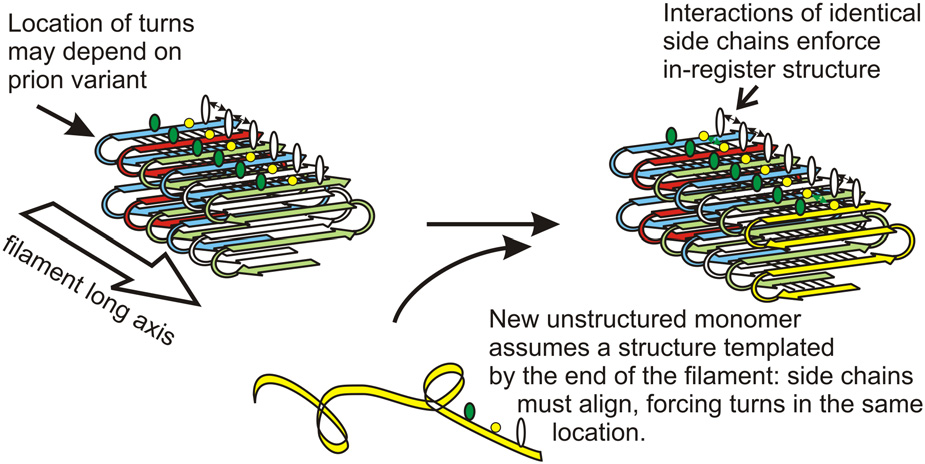
Protein conformation templating mechanism. The in-register parallel amyloid architecture naturally suggests a mechanism for transfer of conformation information from molecules in the filament to those joining the filament. H-bonding or hydrophobic favorable interactions among identical side chains require in-register alignment for the interactions. This directs the monomer joining the end of the filament to have its folds/turns at the same residues as previous molecules in the filament. Different prion variants have folds/turns at different locations, but each is faithfully propagated by this mechanism. Modified from [6].

Solid-state nuclear magnetic resonance (NMR) measurements and filament mass per length determinations show that amyloid filaments of the prion domains (the amyloid-forming parts) of the yeast prion proteins Sup35p, Ure2p, and Rnq1p have a folded, in-register, parallel β-sheet architecture (Fig. 1) (e.g., [15,16]). For Sup35p, we have evidence for the location of some folds [16]. This means that for each residue, there is a line of identical side chains along the long axis of the fiber. Positive interactions among these side chains (hydrophobic interactions for LVIYFWM or hydrogen bonding for QNST; there are very few charged residues) can only happen if the structure is in-register, and so serve to keep it in-register. Filament elongation occurs by addition of monomers [17], and the same positive interactions force a new monomer joining the end of the filament to align with monomers already in the filament, and so have its turns (locations of the folds in the sheet) in the same locations, and the same extent of β-sheet (Fig. 1) [6,18]. In this way, the protein molecules template their conformation and impose it on monomers joining the ends of the filament, just as DNA templates its sequence [6,18].

## The Anti-prion Proteins Btn2 and Cur1

A search for yeast proteins whose overproduction cures the [URE3–1] prion produced two paralogs, Btn2p and Cur1p [19]. Early in the process of curing of [URE3–1] by overproduction of Btn2p, the aggregates of Ure2p, normally scattered in the cytoplasm in [URE3] strains [20], were concentrated at a single locus in the cell, coincident with Btn2p, also in a single site in the cell [19]. This suggests that the overproduced Btn2p sequesters prion aggregates at one site in the cell so that on division, only one of the two daughter cells gets aggregates and the other is cured. In a double mutant *btn2*Δ *cur1*Δ strain, the number of infectious aggregates (“seeds”) is significantly greater than in an isogenic wild-type cell suggesting that Btn2p and Cur1p are sequestering aggregates even at their normal levels [19].

**Fig 2:**
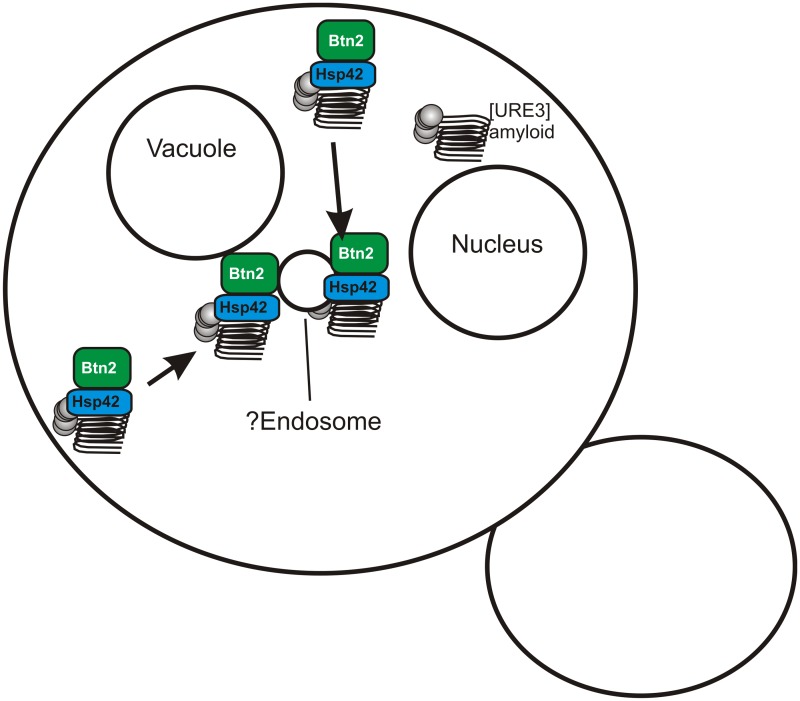
Btn2p sequesters prion amyloid filaments, curing the prion. Btn2p gathers filaments of Ure2p amyloid (the [URE3] prion) to a single site in the cell, possibly the endosome. If the prion has a low seed number then even the normal levels of Btn2p are sufficient to sequester nearly all of the seeds, so that daughter cells without seeds, and therefore cured of the prion, are often produced.

Recently, we generated an array of [URE3]s in a *btn2*Δ *cur1*Δ strain, and found that nearly all such prion isolates were cured by restoration of just the normal levels of either Btn2p or Cur1p (although curing was most efficient if both were restored) [21]. Direct measurements showed that prion variants cured by normal levels of Btn2p or Cur1p had seed numbers several fold lower than variants that were only cured by overproduction of one or the other of these proteins [21]. These “Btn2-Cur1-hypersensitive” variants ([URE3-bcs]) were somewhat unstable and often converted to variants only cured by overproduction of Btn2p or Cur1p. Such less sensitive prion “mutants” had a much higher seed number than their parent. These results support our prion sequestration model of curing: low seed number variants can be well-sequestered by normal levels of Btn2, while higher seed number variants need overproduced Btn2 to be cured (Fig. 2).

Yeast have an array of methods of dealing with protein aggregates, whether amyloid or non-amyloid, including degradation by the ubiquitin-proteasome system, the autophagy system, aggresomes in mammalian cells, chaperones to resolubilize, and others. Inducing autophagy does not cure [URE3], nor does blocking autophagy prevent overproduced Btn2 or Cur1 from curing [URE3] [19]. Bmh1p is necessary for the collection of polyQ aggregates at a special site in yeast, possibly comparable to the aggresome [22], but *bmh1*Δ does not interfere with curing of [URE3] by overproduced Btn2 or Cur1 [21]. The E3 ubiquitin ligases San1p and Ubr1p responsible for marking cytoplasmic proteins for degradation in nuclear proteasomes [23] are not needed for [URE3] curing by overproduced Btn2 or Cur1 [21].

Hsp42, a small heat shock protein, was found to bring several non-amyloid aggregates to a site peripheral to the nucleus, and to be necessary for their collection at that site [24]. Hsp42 interacts with Btn2 and these two proteins colocalize with each other and non-amyloid aggregated proteins [25,26]. We found that Hsp42 is needed for curing of [URE3] by overproduction of Btn2p, and overproduction of Hsp42 itself cures [URE3]. Hsp42 curing of [URE3] requires Cur1p [21]. Evidently Btn2p, Cur1p, and Hsp42 work together in this prion-curing process, apparently by sequestering prion aggregates.

The Btn2/Cur1 system(s) selects the small minority of [URE3] variants that are not cured by normal levels of these proteins and, accordingly, the frequency of spontaneous [URE3] is five-fold higher in a *btn2*Δ *cur1*Δ strain than in wild type [21]. Thus, cells are quite effective in eliminating this prion, suggesting that the cells do not consider having this prion to be a “good thing.”

## Conclusions

Remarkably, a single protein sequence can be the basis of many different protein variants, based on different (but as yet not precisely defined) conformations of the protein in the amyloid (e.g., [3,27]). The folded parallel in-register β-sheet architecture of yeast prion amyloids naturally suggests a templating mechanism that explains this remarkable fact [18]. Just as DNA can be a gene by templating its sequence, proteins can be genes by templating their conformation.

While an array of methods have been found to cure yeast prions by over- or underproduction of various chaperones and other proteins and by various conditions (reviewed in [28]), Btn2 and Cur1 cure the [URE3] prion at normal expression levels, indicating that this is a cellular anti-prion system. The homology of Btn2p to human HOOK proteins, a family that includes the aggresome-promoting protein HOOK2, suggests that information gleaned from the yeast systems will have application in efforts to control human prions and amyloidoses.
